# Erratum: Lee, J.G., et al. The Neuroprotective Effects of Melatonin: Possible Role in the Pathophysiology of Neuropsychiatric Disease. *Brain Sciences* 2019, *9(10)*, 285

**DOI:** 10.3390/brainsci9120341

**Published:** 2019-11-25

**Authors:** Jung Goo Lee, Young Sup Woo, Sung Woo Park, Dae-Hyun Seog, Mi Kyoung Seo, Won-Myong Bahk

**Affiliations:** 1 Department of Psychiatry, College of Medicine, Haeundae Paik Hospital, Inje University, Busan 47392, Korea; iybihwc@inje.ac.kr; 2 Paik Institute for Clinical Research, Department of Health Science and Technology, Graduate School, Inje University, Busan 47392, Korea; swpark@inje.ac.kr; 3 Department of Psychiatry, College of Medicine, The Catholic University of Korea, Seoul 07345, Korea; youngwoo@catholic.ac.kr; 4 Department of Convergence Biomedical Science, College of Medicine, Inje University, Busan 47392, Korea; 5 Department of Biochemistry, College of Medicine, Inje University, Busan 47392, Korea; daehyun@inje.ac.kr; 6 Paik Institute for Clinical Research, Inje University, Busan 47392, Korea; banaba66@inje.ac.kr

The authors wish to make an erratum to the published version of their paper [1]. The authors would like to acknowledge the use of adapted versions of two figures from a recent review by Alghamdi [2] and have added the citation to Figures 2 and 3 in their work [1]. The authors have also added a citation of this recent review by Alghamdi [2] within Section 3.1. of their paper [1].
